# Network Structure and Luminescent Properties of ZnO–B_2_O_3_–Bi_2_O_3_–WO_3_:Eu^3+^ Glasses

**DOI:** 10.3390/ma16206779

**Published:** 2023-10-20

**Authors:** Aneliya Yordanova, Margarita Milanova, Reni Iordanova, Margit Fabian, Lyubomir Aleksandrov, Petia Petrova

**Affiliations:** 1 Institute of General and Inorganic Chemistry, Bulgarian Academy of Sciences, Acad. G. Bonchev Str., bld. 11, 1113 Sofia, Bulgaria; a.yordanova@svr.igic.bas.bg (A.Y.); reni@svr.igic.bas.bg (R.I.); lubomir@svr.igic.bas.bg (L.A.); 2 Centre for Energy Research, 29-33 Konkoly Thege Street, 1121 Budapest, Hungary; fabian.margit@ek.hun-ren.hu; 3 Institute of Optical Materials and Technologies “Acad. Jordan Malinowski”, Bulgarian Academy of Sciences, Acad. G. Bonchev Str., bld. 109, 1113 Sofia, Bulgaria; petia@iomt.bas.bg

**Keywords:** glass structure, europium, IR, photoluminescence, density

## Abstract

In this study, we investigated the influence of Bi_2_O_3_ and WO_3_ on both structure and optical properties of 50ZnO:(49 − x)B_2_O_3_:1Bi_2_O_3_:xWO_3_; x = 1, 5, 10 glasses doped with 0.5 mol% Eu_2_O_3_. IR spectroscopy revealed the presence of trigonal BØ_3_ units connecting superstructural groups, [BØ_2_O]^−^ metaborate groups, tetrahedral BØ_4_^−^ units in superstructural groupings (Ø = bridging oxygen atom), borate triangles with nonbridging oxygen atoms, [WO_4_]^2−^ tetrahedral, and octahedral WO_6_ species. Neutron diffraction experimental data were simulated by reverse Monte Carlo modeling. The atomic distances and coordination numbers were established, confirming the short-range order found by IR spectra. The synthesized glasses were characterized by red emission at 612 nm. All findings suggest that Eu^3+^ doped zinc borate glasses containing both WO_3_ and Bi_2_O_3_ have the potential to serve as a substitute for red phosphor with high color purity.

## 1. Introduction

Currently, white-light-emitting diodes are being investigated extensively as the next-generation solid-state light source owing to the advantages they bring to the table, including safety, an environmentally friendly nature, high stability, low power consumption, and long operational lifetime [1]. One of the ways in which white light can be obtained is by combining tri-color phosphors, such as ZnS:Cu^+^, Al^3+^ (green) [2], BaMgAl_10_O_17_:Eu^2+^ (blue) [3], and Y_2_O_2_S: Eu^3+^ (red) [4], coated on InGaN-based LED chip, emitting around 400 nm (near-UV). However, the red-emitting phosphor shows lower efficiency (eight times lower) compared to the blue and green phosphors, as well as exhibiting chemical instability under UV radiation which may cause environmental pollution due to the release of sulfide gas. The other commercially applied red phosphors, such as Y_2_O_3_:Eu^3+^ and YVO_4_:Eu^3+^, also cannot achieve high emission efficiency [5]. Therefore, red-emitting phosphor with chemical and thermal stability and high efficiency upon near-UV excitation remains to be found. 

Europium (III) ion is being considered as a suitable activator for red emission, resulting from its ^5^D_0_ → ^7^F_j_ (j = 0–4) transitions in the visible range [6]. Unfortunately, Eu^3+^-doped materials cannot be efficiently excited by the present LED chips, because its excitation peaks are weak in nature due to parity-forbidden f–f transitions. Searching for host materials that can overcome the weak Eu^3+^ absorptions is important for achieving high excitation and emission efficiency of the red luminescence. A possibility is introducing sensitizers into the host composition, such as Ce^3+^, Bi^3+^, Tb^3+^, etc. [7,8]. It is well known that the luminescent properties of Eu^3+^-doped materials can be modified by changing the host structure and composition. Glass materials are suitable matrixes for doping with lanthanide ions due to their chemical stability, high optical homogeneity, absence of absorbing particles, and low nonlinear refractive indices. Among many potential glass materials for luminescence applications, binary ZnO–B_2_O_3_ glasses have been attracting continuous scientific interest. Homogeneous binary zinc–borate glasses are formed in a very narrow range of compositions because of the existence of a very large region of immiscibility of two liquids in a ZnO–B_2_O_3_ system [9]. However, these glasses are characterized by good chemical and thermal stability, high mechanical strength, low dispersion, and low glass transition temperature. They possess high transparency (up to 90%) from the visible to mid-infrared region of the spectrum [10]. Eu^3+^-doped zinc borate glasses yield very strong orange/red photoluminescence by UV excitation, especially for low europium concentrations (<10^19^ cm^−^^3^) [11]. It has been reported that the addition of WO_3_ and/or Bi_2_O_3_ to ZnO–B_2_O_3_ glasses induces the expansion of the glass-forming region and also lowers the phonon energy [12,13]. In our recent works, we reported, for the first time, the preparation of tungsten-containing ZnO–B_2_O_3_ glasses doped with Eu^3+^ active ion and their luminescent properties [14,15]. The obtained results from glass structure, physical, thermal, and optical properties indicate the suitability of the 50ZnO:40B_2_O_3_:10WO_3_ glass network for the luminescence performance of Eu^3+^ ions. The positive effect of the addition of WO_3_ on the luminescence intensity is proven by the stronger Eu^3+^ emission of the zinc–borate glass containing WO_3_ compared to the WO_3_-free zinc–borate glass, a phenomenon engendered mainly by the energy transfer from tungstate groups to the Eu^3+^ ions (sensitizing effect). The most intense luminescence peak observed at 612 nm and the high-integrated emission intensity ratio (R) of the ^5^D_0_ → ^7^F_2_/^5^D_0_ → ^7^F_1_ transitions at 612 nm and 590 nm of 5.77 suggest that the glasses have the potential for red emission materials. 

Another desirable component for luminescent glass hosts is Bi_2_O_3_ oxide, commonly used as an activator, emitting in the spectral region of 380–700 nm due to ^3^P_1_ → ^1^S_0_ transition upon NUV excitation. Among many studies, the Bi^3+^ ion is also recognized as a favored sensitizer, which can greatly enhance the luminescence of the rare-earth ions (Eu^3+^, Sm^3+^, Tb^3+^) through resonant energy transfer [16,17]. High bright red emission in Eu^3+^ containing zinc–borate glasses codoped with Bi^3+^ was observed, enhanced by 346 nm excitation (^1^S_0_-^3^P_1_ of Bi^3+^ ions) due to the sensitization effect of Bi^3+^ codopant [18]. Zinc bismuth borate glasses doped with different Eu^3+^ concentrations (1, 3, 5, 7, and 9 mol%) were prepared, and the systematic analysis of the results suggested that the glass doped with a Eu^3+^ concentration of 5 mol% is suitable for LED and display device applications [19]. 

More recently, we prepared zinc–borate glasses modified with Bi_2_O_3_. Bulk, transparent, dark brownish glasses with composition 50ZnO:(40 − x)B_2_O_3_:10Bi_2_O_3_:0.5Eu_2_O_3_:xWO_3_, x = 0 and 0.5, were synthesized. The obtained structural and optical data indicate that a zinc–borate glass network containing Bi_2_O_3_ provides highly asymmetric sites of Eu^3+^ ions, leading to high emission intensity. Moreover, the presence of WO_3_ also leads to the increase in emission intensity of the rare-earth Eu^3+^ ion, as a result of the nonradiative energy transfer from the glass host to the active ion [20]. These data above show that the ZnO–B_2_O_3_ glass system containing both bismuth and tungstate oxides is a particularly interesting host for the europium ions in red phosphors applications. 

Here, we continued our investigations by preparing such a glass composition with increasing WO_3_ content and with the addition of low Bi_2_O_3_ concentrations (1 mol%) in order to meet the requirement of colorless glasses for optical application. The aim was to obtain bulk, colorless glasses with compositions 50ZnO:(49 − x)B_2_O_3_:1Bi_2_O_3_:xWO_3_:0.5Eu_2_O_3_, x = 1, 5, and 10 mol%, and to establish the influence of Bi_2_O_3_ and WO_3_ on glass formation, structure, and optical properties.

## 2. Materials and Methods

### 2.1. Sample Preparation

Glasses of the compositions in mol% 50ZnO:(49 − x)B_2_O_3_:1Bi_2_O_3_:xWO_3_; x = 1, 5, 10, doped with 0.5 mol% Eu_2_O_3_ were obtained by applying the melt quenching method, using reagent-grade ZnO (Merck KGaA, Amsterdam, The Netherlands), WO_3_ (Merck KGaA, Darmstadt, Germany), B_2_O_3_ (SIGMA-ALDRICH, St. Louis, MO, USA), Bi_2_O_3_ (Alfa Aesar, Karlsruhe, Germany), and Eu_2_O_3_ (SIGMA-ALDRICH, St. Louis, MO, USA) as raw materials. B_2_O_3_ enriched with ^11^B isotope (99.6%) was used in order to avoid the high neutron absorption cross-section of the ^10^B isotope. Further in the text, samples’ names are abbreviated to ZBBW1:Eu, ZBBW5:Eu, and ZBBW10:Eu, where the number refers to the WO_3_ content in the compositions. The homogenized batches were melted at 1200 °C for 20 min in a platinum crucible in air. The melts were cast into graphite molds to obtain bulk glass samples. Glasses obtained by us earlier with the same compositions (50ZnO:(50 − x)B_2_O_3_:xWO_3_; x = 1, 5, 10) doped with 0.5 mol% Eu_2_O_3_ without Bi_2_O_3_, and denoted as ZBW1:Eu, ZBW5:Eu, and ZBW10:Eu, were also included here in order to establish the influence of bismuth addition on the structure and luminescence properties of the new glass compositions. The glasses without Bi_2_O_3_ containing 1 and 5 mol% WO_3_ were reported in the 6^th^ International Conference on Optics, Photonics, and Lasers (OPAL’ 2023), while the glass with the highest WO_3_ amount of 10 mol% was represented in ref. [15].

### 2.2. Characterization Techniques

The phase formation of the samples was established by X-ray phase analysis with a Bruker D8 advance diffractometer, Karlsruhe, Germany, using Cu K_α_ radiation in the 10 < 2θ < 60 range. The thermal stability of the obtained glasses was examined by differential scanning calorimetry (DSC) with a Netzsch 404 F3 Pegasus instrument, Selb, Germany, in the temperature range 25–750 °C at a heating rate of 10 K/min in an argon atmosphere. The density of the obtained glasses at room temperature was measured by the Archimedes principle using toluene (ρ = 0.867 g/cm^3^) as an immersion liquid on a Mettler Toledo electronic balance of sensitivity 10^−4^ g. The IR spectra of the glasses were measured using the KBr pellet technique on a Nicolet-320 FTIR spectrometer, Madison, WI, USA, with a resolution of ±4 cm^−1^, by collecting 64 scans in the range 1600–400 cm^−1^. A random error in the center of the IR bands was found to be ±3 cm^−1^. The EPR analyses were carried out in the temperature range 120–295 K in X band at frequency 9.4 GHz on a spectrometer Bruker EMX Premium, Karlsruhe, Germany. Optical absorption spectra (UV–VIS–NIR) in the range 190–1500 nm were obtained with an error ˂ 1% using a commercial double-beam spectrometer (UV-3102PC, Shimadzu, Kyoto, Japan). Photoluminescence (PL) excitation and emission spectra at room temperature for all glasses were measured with a Spectrofluorometer FluoroLog3–22, Horiba JobinYvon, Longjumeau, France. Neutron diffraction measurements were carried out in the momentum transfer range, Q = 0.45–9.8 Å^−1^, for 24 h using neutrons of de Broglie wavelength, λ = 1.069 Å, at the 2-axis PSD diffractometer of Budapest Neutron Centre. The powder glass samples were mounted in a thin-walled cylindrical vanadium can with a diameter of 8 mm for the neutron diffraction experiments. The neutron diffraction data were corrected for detector efficiency, background scattering, and absorption effects, and normalized with vanadium [21]. The total structure factor, *S*(*Q*), was calculated using local software packages. 

### 2.3. The Reverse Monte Carlo Simulation

Reverse Monte Carlo (RMC) simulations were performed on neutron diffraction datasets of the experimental total structure factor, *S*(*Q*), to determine the short-range structural properties of glasses by using RMC++ software (https://www.szfki.hu/~nphys/rmc++/downloads.html, accessed on 15 October 2023) [22]. The RMC technique minimizes the squared difference between the experimental *S*(*Q*) and the simulated one from a 3-dimensional atomic configuration by using the following equations:

(1) $$S(Q)=\sum_{i,j}^{k}w_{ij}S_{ij}(Q)$$

(2) $$S_{ij}(Q)=1+\frac{4\pi \rho_{0}}{Q}\int_{0}^{r_{max}}r\left[g_{ij}(r)-1\right]\sin Qr\;dr$$

(3) $$w_{ij}=\frac{c_{i}c_{j}b_{i}b_{j}}{{\left[\sum_{i,j}^{k}c_{i}b_{j}\right]}^{2}}$$

where *c_i_* and *b_i_* are the molar fraction and coherent neutron scattering length for atoms of type i, the *S_ij_*(*Q*) denotes the partial structure factors, and *w_ij_* are the neutron scattering weight factors for the 21 atomic pairs for the ZBBW:Eu series (explanation: *k* = 5, thus *k*(*k* + 1)/2 = 15 different atomic pairs are present). RMC simulations were used to generate partial atomic pair correlation function, *g_ij_*(*r*), and coordination number distributions. The simulation was started with an initial random configuration by building a box that contained 10.000 atoms of Zn, B, Bi, W, Eu, and O, with the atomic density, *ρ*_o_, values of 0.0947 Å^−^^3^, 0.0919 Å^−^^3^, and 0.0888 Å^−^^3^ for the samples ZBBW1:Eu, ZBBW5:Eu, and ZBBW10:Eu, respectively. The RMC model box lengths for the three samples were 23.63 Å, 23.87 Å, and 24.14 Å for ZBBW1:Eu, ZBBW5:Eu, and ZBBW10:Eu samples, respectively.

In the RMC simulation procedure, constraints were used for the minimum interatomic distances between atom pairs (cut-off distances) to avoid unreasonable atom contacts. For each sample, about fifty RMC configurations were obtained with more than 2,600,000 accepted configurations of atoms.

## 3. Results

### 3.1. XRD Analysis and DSC Studies

**Figure 1:**
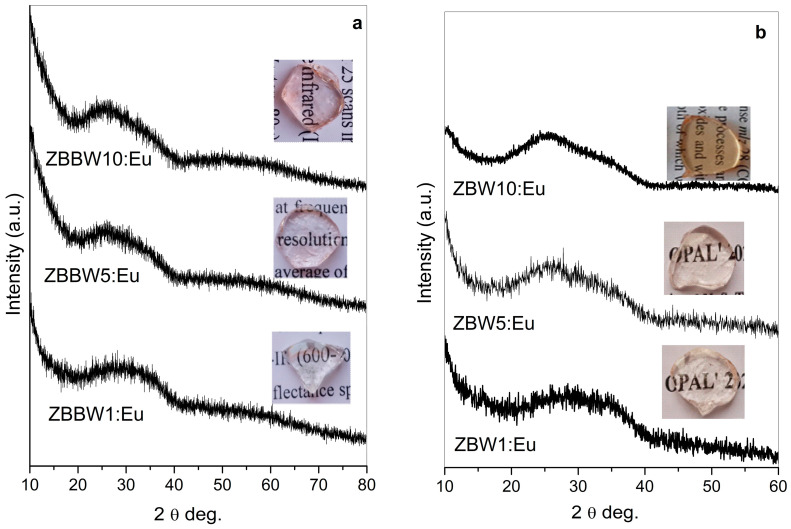
XRD patterns of (**a**) 50ZnO:(49 − x)B_2_O_3_:1Bi_2_O_3_:xWO_3_:0.5Eu_2_O_3_, x = 1, 5, and 10 mol%; (**b**) 50ZnO:(50 − x)B_2_O_3_:xWO_3_:0.5Eu_2_O_3_, x = 1, 5, and 10 mol%.

Bulk, transparent, slightly colored glasses (insets, Figure 1a) of 50ZnO:(49 − x)B_2_O_3_:1Bi_2_O_3_:xWO_3_:0.5Eu_2_O_3_, x = 1, 5, and 10 mol%, were obtained in this study. The measured X-ray diffraction patterns are shown in Figure 1a, and confirm the amorphous nature of the prepared materials. Glasses without Bi_2_O_3_ (50ZnO:(50 − x)B_2_O_3_:xWO_3_:0.5Eu, x = 1, 5, and 10 mol%) were obtained earlier. The XRD patterns of the glass samples having 1 and 5 mol% WO_3_ were present in the 6th International Conference on Optics, Photonics, and Lasers (OPAL’ 2023). The photograph of the glass with the highest WO_3_ amount of 10 mol% was represented in ref. [15]. 

**Figure 2:**
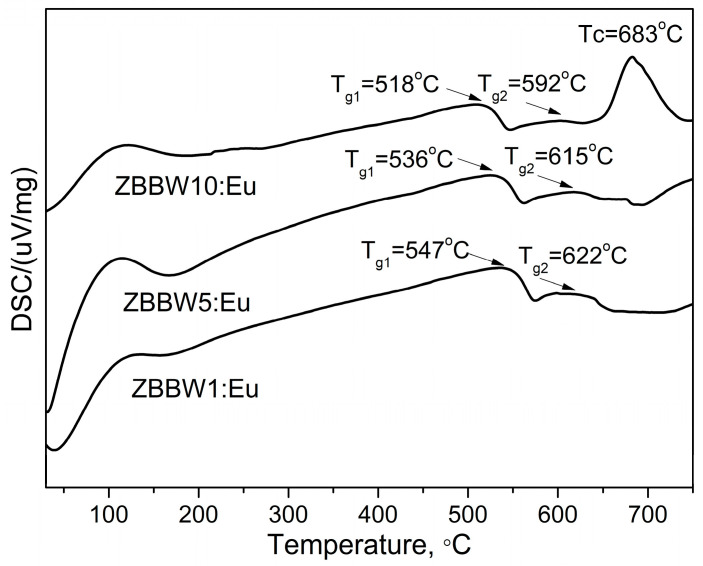
DSC curves of glasses 50ZnO:(49 − x)B_2_O_3_:1Bi_2_O_3_:xWO_3_:0.5Eu_2_O_3_, x = 1, 5, and 10 mol%.

DSC curves of the glass samples 50ZnO:(49 − x)B_2_O_3_:1Bi_2_O_3_:xWO_3_:0.5Eu_2_O_3_, x = 1, 5, and 10 mol%, obtained are presented in Figure 2. Glasses are characterized with two humps corresponding to the two glass transition temperatures, T_g1_ and T_g2_. The two glass transition effects observed are connected with the presence of two amorphous phases with different compositions in the investigated glasses. In the DSC curve of the glass having the highest WO_3_ concentration of 10 mol%, an exothermic peak due to the glass crystallization at temperature Tc = 683 °C was observed. For the glasses containing lower WO_3_ concentration of 5 and 1 mol%, glass crystallization effects did not appear, evidencing that these glasses possess higher thermal stability, which decreases with increasing WO_3_ content. The DSC analysis shows that the thermal parameters of glasses present here do not differ significantly from those obtained for the glasses with the similar compositions without Bi_2_O_3_ reported in Ref. [15]. However, the thermal stability of glasses containing 1 mol% Bi_2_O_3_ was slightly lowered, most probably because of the increased structural heterogeneity and, hence, higher crystallization ability of the composition.

### 3.2. IR Spectral Analysis

**Figure 3:**
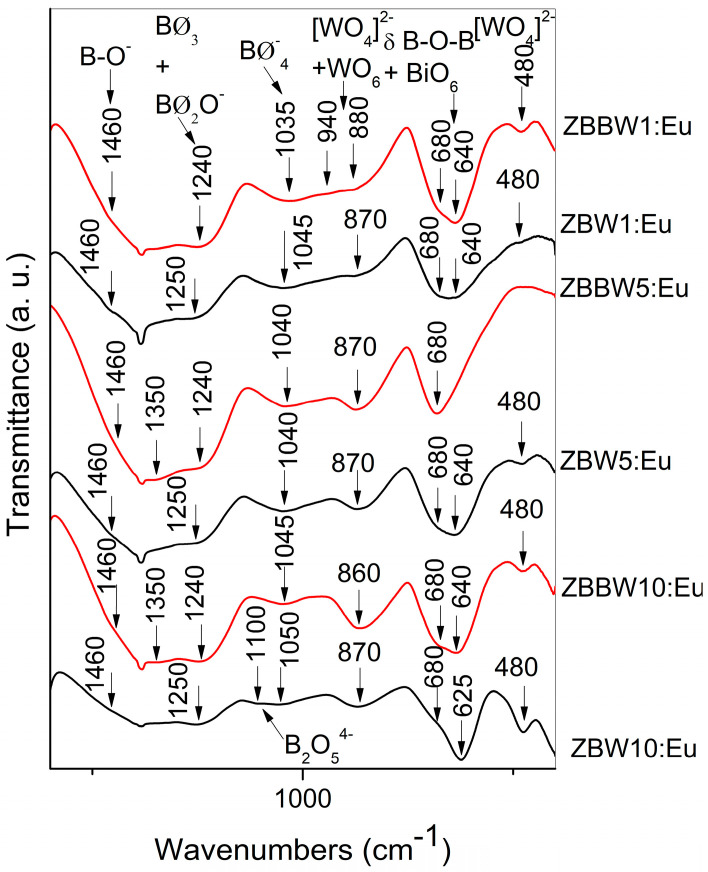
IR spectra of 50ZnO:(49 − x)B_2_O_3_:1Bi_2_O_3_:xWO_3_:0.5Eu_2_O_3_, x = 1, 5, and 10 mol% (in red); and 50ZnO:(50 − x)B_2_O_3_:xWO_3_:0.5Eu_2_O_3_, x = 1, 5, and 10 mol% (in black).

Structural information of the studied glasses was obtained by comparative analysis of IR spectra of glasses with the same compositions without Bi_2_O_3_ and containing 1 mol% Bi_2_O_3_, which are shown in Figure 3. 

The IR spectra of Bi_2_O_3_-free glasses with the lower WO_3_ concentration of 5 and 1 mol% were previously reported by us in the 6th International Conference on Optics, Photonics, and Lasers (OPAL’ 2023), while the glass with the highest WO_3_ amount of 10 mol% was discussed earlier in ref. [15]. The IR spectra of the glasses without Bi_2_O_3_ contain bands characteristic to the metaborate groups BØ_2_O^−^ (shoulder at 1460 cm^−1^; band at 625–640 cm^−1^), pyroborate dimmers, B_2_O_5_^4−^ (weak band at 1110 cm^−1^; band at 625–640 cm^−1^), and superstructural groupings with BØ_3_ and BØ_4_^−^ species (bands at 1250 cm^−1^ and at 1040–1050 cm^−1^, band at 680 cm^−1^) [14,23,24,25]. There are also WO_6_ (bands at 870 cm^−1^ and at 680 cm^−1^) and [WO_4_]^2−^ tetrahedra (band at 480 cm^−1^) [23]. The addition of Bi_2_O_3_ to the glass compositions causes the BO_3_ →BO_4_ transformation, resulting in the increase in the number of superstructural units (increased intensity of the bands at 1240 and 1045–1035 cm^−1^ and band at 680 cm^−1^) [23]. For both the glass series, a partial [WO_4_]^2−^ → WO_6_ transformation with increasing WO_3_ concentration occurs, manifested by the disappearance of the bands at 940 cm^−1^ and 880 cm^−1^ (ν_3_[WO_4_]^2−^), and formation of an intense and well-formed band at 860–870 cm^−1^ (νWO_6_). The tungstate octahedral species sharing common corners (W–O–W) and edges (W_2_O_2_) are supposed, having in mind the structural and IR data of crystalline Bi_2_WO_6_, Bi_2_W_2_O_9_, and ZnWO_4_ phases [26,27,28,29,30,31]. These compounds consist of corner- or edge-shared WO_6_, whose IR spectra contain strong bands situated in the same spectral regions, from 870–800 cm^−1^ and 700–600 cm^−1^. Tungstate and borate species are charge-balanced by Bi^3+^, Zn^2+^, and Eu^3+^ ions via Bi–O–W, B–O–Bi, Zn–O–W, Zn–O–B, Eu–O–W, and Eu–O–B bonding. Additionally, the new high frequency band at 1350 cm^−1^ observed in glasses having higher WO_3_ of 5 and 10 mol% (Figure 2, ZBBW5:Eu and ZBBW10:Eu) is attributed to stretching of B–O^−^ bonds in BØ_2_O^−^ triangles, and its presence suggests stronger interaction between Bi^3+^ ions and nonbridging oxygens [32]. Bi^3+^ ions are incorporated in the structure of investigated glasses as BiO_6_ octahedra (bands at 640 and at 480 cm^−1^). Thus, the addition of Bi_2_O_3_ to glasses 50ZnO:(49 − x)B_2_O_3_:xWO_3_:0.5Eu_2_O_3_, x = 1, 5, and 10 mol%, leads to the formation of more stable and reticulated glass structure, compared with the glasses with the same composition without Bi_2_O_3_. The Zn^2+^ ions generally participate in the borate glasses as ZnO_4_ tetrahedra with characteristic Zn^2+^ motion at 225 cm^−1^ [33]. The frequency of the Eu–O vibration, ν(Eu–O), has been measured at about 280 cm^−1^ for glasses (1 − 2x)Eu_2_O_3_ − x(SrO − B_2_O_3_) [34]. 

The detailed assignments of the bands observed in the IR spectra of the present glasses are summarized in Table 1.

### 3.3. Density, Molar Volume, Oxygen Packing Density, and Oxygen Molar Volume

A structural information of two series of glasses was also gained by density (ρ_g_) measurement, on which basis the values of several physical parameters listed in Table 2 (molar volume (V_m_), oxygen molar volume (V_o_), and oxygen packing density (OPD)) are evaluated, using the conventional formulae [37]. Bi_2_O_3_ containing glasses are characterized with the higher density as compared with the respective Bi_2_O_3_-free glasses because of the replacement of lighter B_2_O_3_ (molecular weight 69.62 g/mol) with heavier Bi_2_O_3_ (molecular weight 465.96 g/mol). The V_m_ and V_o_ values of glasses having 1 mol% Bi_2_O_3_ are lower, while their OPD values are higher as compared with the values of the same parameters established for the glasses without Bi_2_O_3_, evidencing better packing and bonding in the Bi_2_O_3_-containing glass network and lower number of nonbridging oxygens (NBOs) [38].

### 3.4. RMC Modeling and Results

**Figure 4:**
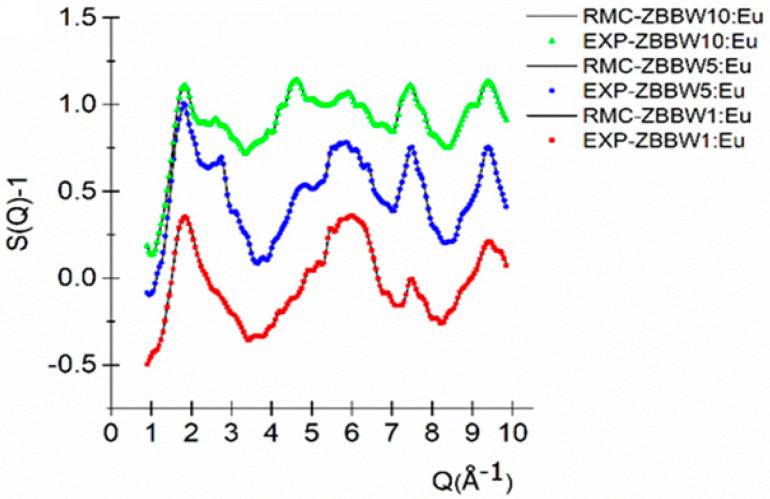
Experimental (color) and RMC (black line)-simulated neutron scattering structure factors for 50ZnO:(49 − x)B_2_O_3_:1Bi_2_O_3_:xWO_3_:0.5Eu_2_O_3_, x = 1, 5, and 10 mol% glasses.

The RMC technique provided an excellent fit of the simulated structure factors (S(Q)-1) with the experimental one for 50ZnO:(49 − x)B_2_O_3_:1Bi_2_O_3_:xWO_3_:0.5Eu_2_O_3_, x = 1, 5, and 10 mol% (Figure 4). 

From the RMC simulation, partial atomic pair-correlation functions, *g_ij_*(*r*), and average coordination number distributions, CN_ij_, were revealed, with good stability and statistics. The Zn–O distribution functions show symmetrical peaks centered in the range of 1.95 ± 0.01 Å (Table 3). 

In function of *g_ij_*(*r*), we specify a range in r over which atoms are counted as neighbors. This can be understood as defining coordination shells. Introducing a *min* point (positions of minimum values on the lower) and *max* point (the upper side of the corresponding peak), these are presented in Table 4, where we present the average coordination numbers (summarized in Table 4).

The average coordination number of the Zn–O was obtained from the RMC analysis, and it was found that Zn^4+^ was tetrahedrally coordinated with oxygens in the glassy network for all studied samples (Table 4). The B–O distribution function showed a relatively broad first neighbor distance at 1.40 ± 0.05 Å, and a slight shoulder at 1.80 ± 0.1 Å appeared in function of concentration in the 50ZnO:(49 − x)B_2_O_3_:1Bi_2_O_3_:xWO_3_:0.5Eu_2_O_3_, x = 1, 5, and 10 mol%, glass samples. The B–O coordination was in the range of 3.48 ± 0.05 to 4.00 ± 0.05, and obtained the changes in BO_3_/BO_4_ ratio. The boron atoms were coordinated mostly by three and four oxygen atoms, forming trigonal BO_3_ and tetrahedral BO_4_ units, in agreement with coordination numbers in SiO_2_–Na_2_O–B_2_O_3_ glasses [39], in MoO_3_–ZnO–B_2_O_3_ glasses [40], and in ZnO–B_2_O_3_–Li_2_O–Al_2_O_3_ glasses [41]. The nearest W–O distances showed characteristic peaks at 1.75 ± 0.05 (Table 3) for both series. The average coordination number of W–O was in a wide range, from 6.20 ± 0.1 to 6.73 ± 0.1 (see Table 3) within the limits of experimental uncertainty. Based on the coordination numbers, we can predict that the W–O network consists of WO_4_ and WO_6_ units. The WO_4_/WO_6_ ratio changed with the WO_3_ concentration, and samples with highest WO_3_ concentration (10 mol%) had mostly WO_6_ units with fewer WO_4_ units. In the case of Bi–O and Eu–O, thanks to the very low Bi_2_O_3_ and Eu_2_O_3_ concentration, it was not relevant to obtain reliable numbers for the coordination.

### 3.5. EPR Spectroscopy

**Figure 5:**
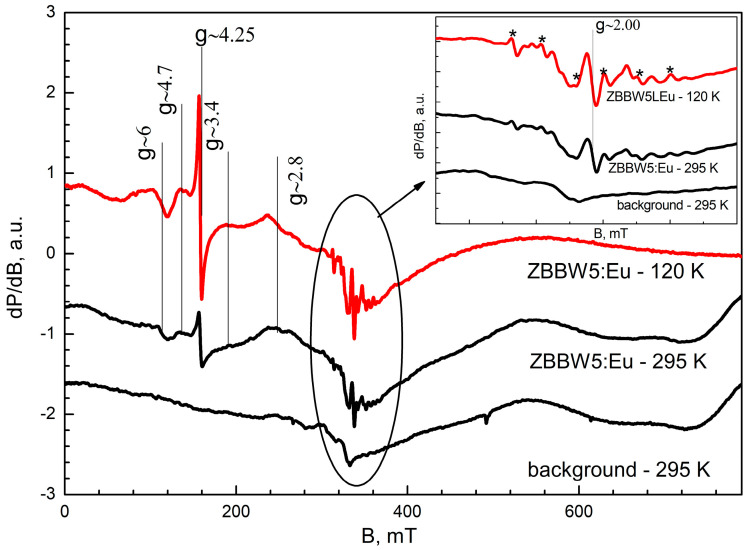
EPR spectra of ZBBW5:Eu, recorded at 120 and 295 K. The quartz tube background is represented at the bottom. EPR measurement conditions: Att = 16 (5 mW); MA = 20.

The EPR analyses of ZBBW5:Eu were carried out at 295 K and 120, and in Figure 5 the obtained spectra are shown. As seen, the spectra contain multiple signals with different intensities and g-factors. The most prominent features are assigned to impurities of isolated Fe^3+^ ions (the signal with *g* = 4.25) and isolated Mn^2+^ ions (six hyperfine structure lines marked with *, inset).

Eu^2+^ ions possess electron spin S = 7/2, and in their EPR spectrum seven signals were observed, corresponding to seven allowed transitions occurring between four Kramers’ doublets (ms = ±1/2, ms = ±3/2, ms= ±5/2, and ms = ±7/2) according to the selection rule [42]. Depending on the zero-field splitting parameters (D, E) and crystal field symmetry, the EPR spectra of Eu^2+^ usually include a part of these signals. In the spectra of ZBBW5:Eu are discernable a set of not-well-resolved and low-intensive signals with g factors about 6.0, 4.7, 3.4, and 2.8 located in the range 0–300 mT. These signals could be assigned to Eu^2+^ ions [42,43] localized in a low-symmetry crystal field with large zero-field splitting (D > hν). 

In addition, the central region of the spectrum shows a signal with *g* = 2.00. The assignment of this signal is somewhat difficult, as it could derive both from Fe^3+^ ion impurities existing in the sample and Gd^3+^ ions in a highly symmetric environment. That is why its attribution remains unclear.

To summarize, the EPR spectra recorded for sample ZBBW5:Eu confirm the presence of Eu^2+^ ions, with their concentration being assessed as extremely low based on the comparison between the background spectrum and the analyzed spectrum.

### 3.6. Luminescent Properties

**Figure 6:**
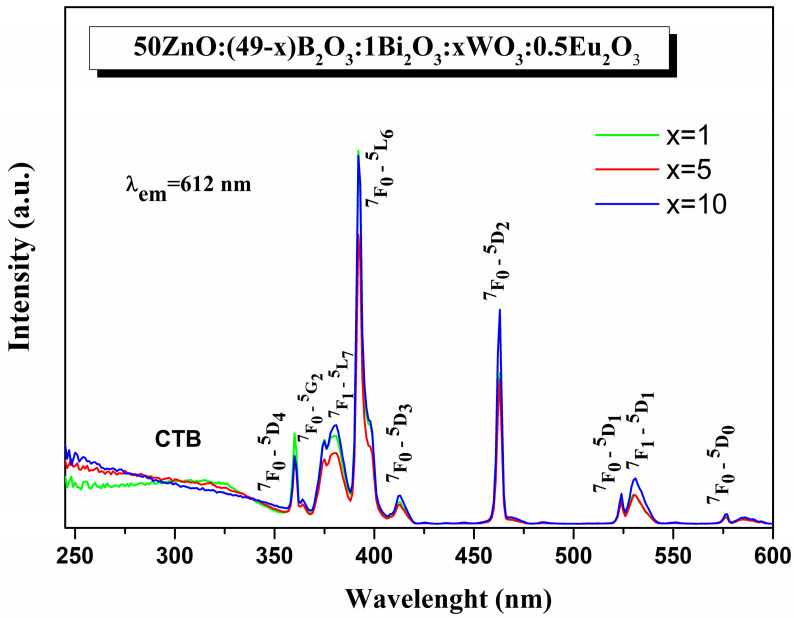
Excitation spectra of 50ZnO:(49 − x)B_2_O_3_:1Bi_2_O_3_:xWO_3_:0.5Eu_2_O_3_ (x = 1, 5, and 10 mol%) glasses.

Figure 6 shows the photoluminescent excitation spectra of Eu^3+^-doped 50ZnO:(49 − x)B_2_O_3_:1Bi_2_O_3_:xWO_3_, x = 1, 5, and 10 mol%, glasses monitoring the ^5^D_0_ → ^7^F_2_ red emission of Eu^3+^ at 612 nm [6]. 

The low-intensive broad band below 350 nm is ascribed to the characteristic absorption of Bi^3+^ (^1^S_0_ → ^3^P_1_) [17], the charge transfer bands (CTB), resulting from energy transitions from O^2−^ to W^6+^ in WO_4_ and WO_6_ groups and O^2−^ to Eu^3+^, as well as the ground 4f state of Eu^3+^ to W^6+^ [6,44,45,46,47,48]. The existence of the excitation band of host lattice absorption at Eu^3+^ emission (612 nm) implies the existence of nonradiative energy transfer from Bi^3+^ and WO_n_ groups to the active rare-earth ion [48,49]. The sharp lines in 350–600 nm range correspond to the f → f intraconfigurational forbidden transitions of Eu^3+^ from the ground state (^7^F_0_) and from the first excited state (^7^F_1_): ^7^F_0_ → ^5^D_4_ (360 nm), ^7^F_0_ → ^5^G_2_ (375 nm), ^7^F_1_ → ^5^L_7_ (381 nm), ^7^F_0_ → ^5^L_6_ (392 nm), ^7^F_0_ → ^5^D_3_ (412 nm), ^7^F_0_ → ^5^D_2_ (463 nm), ^7^F_0_ → ^5^D_1_ (524 nm), ^7^F_1_ → ^5^D_1_ (531 nm), and ^7^F_0_ → ^5^D_0_ (576 nm) [6]. Among them, the electronic transition at 392 nm is the strongest one and was used as an excitation wavelength. Compared to the CTB, the intensity of the narrow f–f lines is stronger. This is favorable for Eu^3+^-doped luminescent materials, since, in general, the intensity of these Eu^3+^ transitions is weak due to the parity-forbidden law. Thus, the obtained glasses can be effectively excited by near-UV and blue light, which is compatible with the present LED chips. 

**Figure 7:**
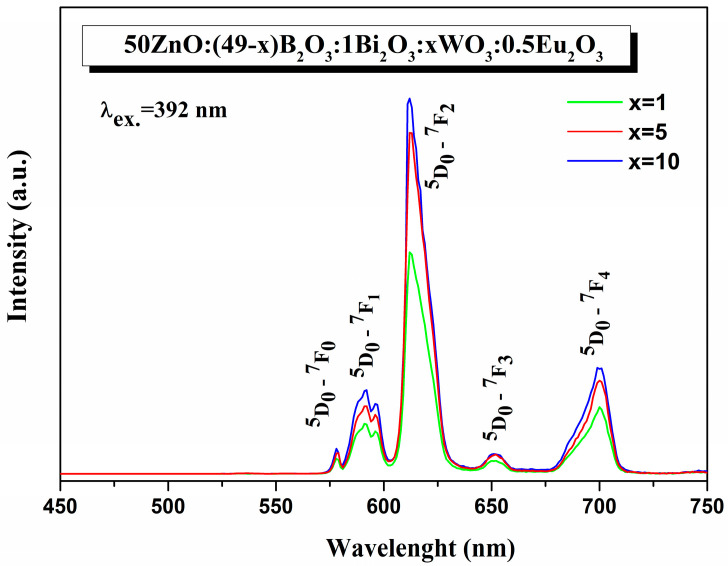
Emission spectra of 50ZnO:(49 − x)B_2_O_3_: 1Bi_2_O_3_:xWO_3_:0.5Eu_2_O_3_ (x = 1, 5, and 10 mol%) glasses.

The emission spectra of Eu^3+^-doped 50ZnO:(49 − x)B_2_O_3_: 1Bi_2_O_3_:xWO_3_, x = 1, 5, and 10 mol%, glasses under the excitation of λ_ex_ = 392 nm consist of five emission peaks centered at 578, 592, 612, 651, and 700 nm, originating from ^5^D_0_ →^7^F_J_ (J = 0, 1, 2, 3, 4) intraconfigurational transitions of Eu^3+^ (Figure 7) [6]. 

**Figure 8:**
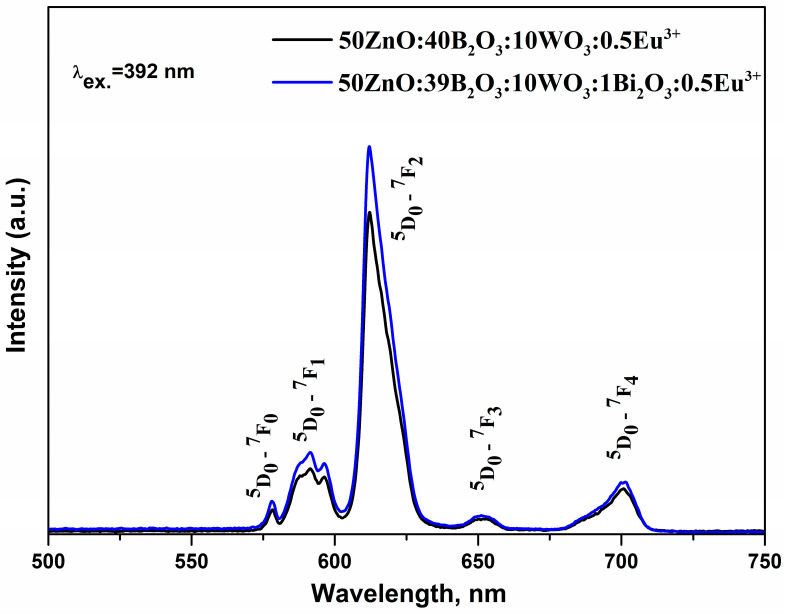
Emission spectra of 50ZnO:(40 − x)B_2_O_3_: xBi_2_O_3_:10WO_3_:0.5Eu_2_O_3_ (x = 0 and 1 mol%).

The characteristic single broad band emission of Bi^3+,^ originating from ^3^P_1_ → ^1^S_0_ transition is located at 380–700 nm [50]. In the same spectral region, we also registered the broad emission band of the WO_n_ group [51]. The general requirement for energy transfer from both WO_3_ and Bi_2_O_3_ to the rare-earth ion is satisfied, i.e., there exists a spectral overlap between the excitation peaks of Eu^3+^ (Figure 6) and the emission band of Bi^3+^ and WO_3_ (Figure 7). As a result, both oxides can act as sensitizers, transferring the emission energy nonradiatively to the activator Eu^3+^ by quenching their luminescence. Moreover, an indication of energy transfer is the absence of characteristics of WO_3_ and Bi_2_O_3_ emission bands [15,20,48]. As can be seen, with the increase of WO_3_ up to 10 mol% (Figure 7) and with the introduction of Bi_2_O_3_ in the 50ZnO:40B_2_O_3_:10WO_3_:0.5Eu_2_O_3_ glass composition (Figure 8), a significant enhancement of the emission intensities was achieved. 

The most intensive emission peak, observed at 612 nm, corresponds to the hypersensitive to the site symmetry electric dipole (ED) transition ^5^D_0_ → ^7^F_2_, while the second-most intensive magnetic dipole (MD) ^5^D_0_ → ^7^F_1_ one is insensitive to the site symmetry and is considered almost constant [6,44,52,53]. The integrated emission intensity ratio (R) of these two transitions ^5^D_0_ → ^7^F_2_/^5^D_0_ → ^7^F_1_ is used to estimate the degree of symmetry around Eu^3+^ ions and the strength of covalence of the europium–oxygen bond. The higher R values indicate more site asymmetry of the rare-earth ion, a high covalency between Eu^3+^ and O^2−^ ions, and an enhanced emission intensity [6,54,55]. The intensity ratios, R, of the present glasses (R = 4.7–5.7) (Table 5) are higher than most of the other reported Eu^3+^-doped glasses and have close values to Eu^3+^:Y_2_O_3_ and Eu^3+^:Y_2_O_2_S, indicating that the synthesized glasses are characterized by a more distorted environment of the Eu^3+^ ion and a high covalent bonding between Eu^3+^ and the surrounding ligands, thus achieving an enhanced Eu^3+^ emission intensity [15,18,20,36,56,57,58,59,60,61,62,63,64].

The R values were found to increase from 4.61 to 5.73 (Table 5) as the WO_3_ concentration raised from 1 to 10 mol%. The incorporation of small amounts of Bi_2_O_3_ (1 mol%) into the glass structure also led to an increase in the asymmetric ratio from 5.57 for the glass 50ZnO:40B_2_O_3_:10WO_3_:0.5Eu_2_O_3_ to 5.73 for the glass 50ZnO:39B_2_O_3_:1Bi_2_O_3_:10WO_3_:0.5Eu_2_O_3_. These R values were much higher as compared to the R value for glass with high Bi_2_O_3_ content (10 mol%) (50ZnO:(40 - x)B_2_O_3_:10Bi_2_O_3_:0.5Eu_2_O_3_:xWO_3_, x = 0 and 0.5) (R = 3.79) [20]. This result shows that we have found an appropriate glass composition for hosting an active rare-earth ion that provide a high Eu^3+^ emission intensity.

## 4. Discussion

In this study, by analyzing the IR spectra of zinc–borate glasses containing 1, 5, and 10 mol% WO_3_, with and without Bi_2_O_3_, it was found that 1 mol% Bi_2_O_3_ leads to an increase in the number of the BO_4_^−^ involved in superstructural groupings and formation of Bi–O–B, Bi–O–W, Zn–O–B, Zn–O–W, Eu–O–W, and Eu–O–B cross-links in the glass structure. With WO_3_ loading in the Bi^3+^-containing glasses, a partial [WO_4_]^2−^ → WO_6_ transformation took place. The tungstate octahedra shared common corners and/or edges, forming W–O–W and W_2_O_2_ bonds. There were also B–O–B bonds with different numbers with the WO_3_ content. The RMC modeling also revealed the presence of both WO_6_ and WO_4_ units and trigonal BO_3_ and tetrahedral BO_4_ units varying in amount with the composition. Thus, the bismuth-containing glasses were characterized by a more reticulated and rigid network that ensures low symmetry sites of Eu^3+^ ions, which is favorable for the luminescence emission of the active Eu^3+^ ion. The structural features revealed by IR analysis agreed well with the measured density and calculated physical parameters. Bi_2_O_3_-containing glasses were characterized with the lower molar and oxygen molar volume and higher oxygen packing density, as compared with Bi_2_O_3_-free glasses with the same compositions, as a result of the formation of highly cross-linked structure and the presence of new mixed bonding with participation of Bi^3+^ ions. A significant enhancement of the Eu^3+^ emission was established in the glasses 50ZnO:(49 − x)B_2_O_3_:1Bi_2_O_3_:xWO_3_:0.5Eu_2_O_3_, (a) x = 1 mol%, (b) x = 5 mol%, (c) x = 10 mol%, in the presence of low Bi_2_O_3_ content (1 mol%) and with the increase of WO_3_ content (up to 10 mol%). The photoluminescent spectra of the new glasses showed an intensive red luminescence at 612 nm as well as a very large value of the luminesce ratio R (over 5), both evidencing that Eu^3+^ ions occupied distorted sites in the created glass network. Particularly, the glass with 10 mol% WO_3_ showed the strongest emission of the active ions as a result of the structural features established and also because of an energy transfer from tungstate and bismuthate groups to the active ion. 

## 5. Conclusions

The results of this investigation show that the zinc borate glass matrix with the simultaneous presence of both Bi_2_O_3_ and WO_3_ is very suitable for implementing the active Eu^3+^ as it possesses a reticulated and rigid glass structure, ensuring a more asymmetrical local structure around Eu^3+^ sites, accordingly yielding a higher luminescence of the incorporated Eu^3+^ ions. On the other hand, both bismuth and tungsten oxides have a synthesizer effect by transferring the emission energy nonradiatively to the activator Eu^3+^**,** which additionally improves its luminesce properties. This suggests that the obtained glasses are potential candidates for red-light-emitting phosphors.

## Figures and Tables

**Table 1:** Infrared bands (in cm^−1^) and their assignments for glasses 50ZnO:(49 − x)B_2_O_3_:1Bi_2_O_3_:xWO_3_:0.5Eu_2_O_3_, x = 1, 5, and 10 mol%.

Infrared Bands Position (cm^−1^)	Assignment	Ref.
475	ν_4_[WO_4_]^2−^ + Bi–O vibrations in the BiO_6_ groups	[32,35]
680	Bending vibrations of B–O–B bonds in superstructural	[32]
640–625	Bending vibrations of B–O–B bonds in meta- and pyroborates + Bi–O vibrations in the BiO_6_ groups	[32]
860–870	νWO_6_	[14,23]
940; 880	ν_3_[WO_4_]^2−^ in distorted tetrahedra	[23]
1050–1035	ν_as_BØ_4_^−^ involved in superstructural units	[14,23]
1100	ν_as_(B–O–B); B–O–B bridge in pyroborate units, B_2_O_5_^4−^	[24,35]
1245	ν_as_(B–O–B); B–O–B bridges connect BO_3_ units + BO_3_ stretch in meta-, pyro-, orthoborate units	[15,36]
1350	ν(B–O^−^) stretch in BØ_2_O^−^ units charge balanced by Bi^3+^	[32]
1460	ν(B–O^−^) stretch in BØ_2_O^−^ units	[23]

**Table 2:** Values of physical parameters of glasses 50ZnO:(49 − x)B_2_O_3_:1Bi_2_O_3_:xWO_3_:0.5Eu_2_O_3_, x = 1, 5, and 10 mol%: density (ρ_g_), molar volume (V_m_), oxygen molar volume (V_o_), oxygen packing density (OPD).

Sample ID	ρ_g_ (g/cm^3^)	V_m_ (cm^3^/mol)	V_o_ (cm^3^/mol)	OPD (g atom/L)
ZBW1:Eu	3.475± 0.002	22.70	11.29	88.55
ZBW5:Eu	3.689± 0.002	23.14	11.51	86.86
ZBW10:Eu *	3.910 ± 0.002 *	23.91 *	11.73 *	84.27 *
ZBBW1:Eu	3.679 ± 0.001	22.57	11.20	89.28
ZBBW5:Eu	3.889 ± 0.005	23.01	11.42	87.57
ZBW10:Eu	4.175 ± 0.001	23.38	11.60	86.18

* The physical parameters of glass ZBW10:Eu were previously reported in Ref. [15].

**Table 3:** Interatomic X–O distances, g_ij_(r), obtained from RMC simulation. The errors are estimated from the reproducibility of various RMC runs.

Title 1	Zn–O *g_ij_*(*r*) (Å)	B–O *g_ij_*(*r*) (Å)	Bi–O *g_ij_*(*r*) (Å)	W–O *g_ij_*(*r*) (Å)	Eu–O *g_ij_*(*r*) (Å)	O–O *g_ij_*(*r*) (Å)
ZBBW1:Eu	1.95 ± 0.01	1.40/1.80 ± 0.05	2.00 ± 0.05	1.75 ± 0.05	2.20 ± 0.05	2.35 ± 0.03
ZBBW5:Eu	1.95 ± 0.01	1.40/1.80 ± 0.05	2.00 ± 0.05	1.75 ± 0.05	2.20 ± 0.05	2.35 ± 0.03
ZBB10:Eu	1.95 ± 0.01	1.40/1.80 ± 0.05	2.00 ± 0.05	1.75 ± 0.05	2.20 ± 0.05	2.35 ± 0.03

**Table 4:** Average coordination numbers, CN_ij_, calculated from RMC simulation. In brackets, the interval is indicated, where the actual coordination number was calculated.

Sample	Zn–O CN_ij_	B–O CN_ij_	W–O CN_ij_	O–O CN_ij_
ZBBW1:Eu	4.01 ± 0.05 (min: 1.80–max: 2.20)	3.90 ± 0.05 (min: 1.20–max: 1.65)	6.20 ± 0.1 (min: 1.65–max: 2.23)	5.63 ± 0.1 (min: 2.20–max: 2.60)
ZBBW5:Eu	3.99 ± 0.05 (min: 1.80–max: 2.20)	3.52 ± 0.05 (min: 1.20–max: 1.65)	6.42 ± 0.1 (min: 1.60–max: 2.25)	5.32 ± 0.1 (min: 2.20–max: 2.60)
ZBBW10:Eu	3.97 ± 0.05 (min: 1.80–max: 2.20)	3.48 ± 0.05 (min: 1.20–max: 1.65)	6.73 ± 0.1 (min: 1.60–max: 2.25)	5.54 ± 0.1 (min: 2.20–max: 2.60)

**Table 5:** Comparison of the luminescence intensity ratio (R) of ^5^D_0_ → ^7^F_2_ to ^5^D_0_ → ^7^F_1_ transition of Eu^3+^-doped oxide glasses.

Glass Composition	R Values	Ref.
50ZnO:48B_2_O_3_:1Bi_2_O_3_:1WO_3_:0.5Eu_2_O_3_	4.61	Present work
50ZnO:44B_2_O_3_:1Bi_2_O_3_:5WO_3_:0.5Eu_2_O_3_	5.04	Present work
50ZnO: 40B_2_O_3_:10WO_3_:0.5Eu_2_O_3_	5.57	[12]
50ZnO:39B_2_O_3_:1Bi_2_O_3_:10WO_3_:0.5Eu_2_O_3_	5.73	Present work
50ZnO:40B_2_O_3_:10WO_3_:xEu_2_O_3_ (0 ≤ x ≤ 10)	4.54–5.77	[15]
50ZnO:40B_2_O_3_:5WO_3_:5Nb_2_O_5_:xEu_2_O_3_ (x = 0, 0·1, 0·5, 1, 2, 5 and 10)	5.09–5.76	[36]
50ZnO:(40 − x)B_2_O_3_:10Bi_2_O_3_:0.5Eu_2_O_3_:xWO_3_, x = 0 and 0.5	3.58; 3.79	[20]
20ZnO:8Al_2_O_3_:(12 − x)Bi_2_O_3_:60B_2_O_3_:xEu_2_O_3_	1.951–2.78	[56]
39.5Li_2_O:59.5SiO_2_:1Eu_2_O_3_	3.20	[57]
4ZnO:3B_2_O_3_ 0.5 ÷ 2.5 mol% Eu^3+^	3.94–2.74	[58]
Eu^3+^: 45B_2_O_3_-5ZnO-49PbO	3.03	[59]
15PbF_2_:25WO_3_:(60 − x)TeO_2_:xEu_2_O_3_ x = 0.1, 0.5, 1.0 and 2.0 mol%	2.37–2.78	[60]
40ZnO:(30 − x) B_2_O_3_:30P_2_O_5_:xEu_2_O_3_ (0.1 ≤ x ≤ 0.9)	2.96–3.65	[61]
60ZnO:(40x)B_2_O_3_:0.2Eu_2_O_3_:xBi_2_O_3_ (x = 0, 0.1, 0.2, 0.5, 1.0)	2.98	[18]
(100 − x):(0.2Bi_2_O_3_–0.8GeO_2_):xEu_2_O_3_ (x = 0.5, 1, 1.5, 2 mol%)	3.94–4.21	[62]
Eu^3+^:Y_2_O_3_	3.8–5.2	[63]
Eu^3+^ doped Y_2_O_2_S	6.45–6.62	[64]

## Data Availability

Not applicable.
